# Prophylactic cholecystectomy: A valuable treatment strategy for cholecystolithiasis after gastric cancer surgery

**DOI:** 10.3389/fonc.2022.897853

**Published:** 2022-09-13

**Authors:** Haipeng Liu, Jie Liu, Wei Xu, Xiao Chen

**Affiliations:** ^1^ Department of General Surgery, Lanzhou University Second Hospital, Lanzhou, China; ^2^ The Second Clinical Medical College, Lanzhou University, Lanzhou, China; ^3^ Key Laboratory of Digestive System Tumors of Gansu Province, Lanzhou University Second Hospital, Lanzhou, China

**Keywords:** gastric cancer, cholecystolithiasis, prophylactic cholecystectomy, cholecystectomy, risk factors

## Abstract

The main treatment for gastric cancer is surgical excision. Gallstones are one of the common postoperative complications of gastric cancer. To avoid the adverse effects of gallstone formation after gastric cancer surgery, we reviewed the causes and risk factors and mechanisms involved in gallstone formation after gastric cancer surgery. The evidence and value regarding prophylactic cholecystectomy (PC) during gastric cancer surgery was also reviewed. Based on previous evidence, we summarized the mechanism and believe that injury or resection of the vagus nerve or changes in intestinal hormone secretion can lead to physiological dysfunction of the gallbladder and Oddi sphincter, and the lithogenic components in the bile are also changed, ultimately leading to CL. Previous studies also have identified many independent risk factors for CL after gastric cancer, such as type of gastrectomy, reconstruction of the digestive tract, degree of lymph node dissection, weight, liver function, sex, age, diabetes and gallbladder volume are closely related to CL development. At present, there are no uniform guidelines for the selection of treatment strategies. As a new treatment strategy, PC has undeniable advantages and is expected to become the standard treatment for CL after gastric cancer in the future. The individualized PC strategy for CL after gastric cancer is the main direction of future research.

## Introduction

Since Majoor and Suren first discussed the phenomenon of cholecystolithiasis (CL) after gastrectomy in 1947 (1), it has been confirmed that CL is one of the major postoperative complications of gastric cancer and that its related complications seriously threaten the life and health of patients (2). The incidence of CL after gastrectomy was reported to be 10-25% (3–8) and has been increasing in recent years (3, 9), being significantly higher than that in the general population (15-25% vs. 2.2-5.0%) (2, 10, 11). However, many issues remain unclear or disputable, such as the main, specific pathophysiological mechanism of gallstone formation after gastric cancer surgery; the risk factors for CL development after gastric cancer surgery; which strategies are the most appropriate treatments for CL after gastric cancer surgery; and whether prophylactic cholecystectomy (PC) should be performed. Because the above issues are controversial, we present a review from seven aspects and put forward our views, as follows.

## Causes and mechanism of CL development after GC surgery

### Vagus nerve disconnection

The vagus nerve is the source of power for the movement of the gallbladder. Studies have confirmed that vagal nerve injury is an important cause of CL after gastric cancer surgery (12), and the 5-year follow-up incidence of CL after vagal nerve dissection is 9%-21% (13). Ihasz et al. (14) found that 34% of the patients in vagectomy group who received gastrectomy had impaired gallbladder systolic function, and 65% had virtually no gallbladder systolic function.

Studies have shown that the vagus nerve trunk has an important regulatory effect on the absorption and metabolism of nutrients (15), and damage to the hepatic and biliary branches of the vagus nerve causes the gallbladder to lose its innervation, changes the dynamic function of the gallbladder, and increases the tension of the Oddi sphincter, which promotes the development of CL (16). Cattey et al. (17) confirmed that the pyloric one-gallbladder reflex was lost after antral gastrectomy, thus inhibiting gallbladder contraction and easily causing CL. This also confirms that the reflex mechanism between pylorus and bile duct is involved in the formation of CL (18).

### Changes in the secretion of intestinal hormone

Cholecystokinin (CCK) can cause gallbladder contraction, Oddi-sphincter and duodenal relaxation (12). The main mechanism of gallbladder contraction is the release of CCK, even after gastrectomy (19). After gastrectomy, the secretory function of gastric mucosa is reduced or even lost; gastric acid is lacking; and after the digestive tract is reconstructed, food is redirected directly into the jejunum without passing through the duodenum, resulting in reduced CCK release (20), thereby promoting the development of CL. In addition, pancreatic hormone, gastrin, glucagon, motility hormone (21), substance P (SP), etc., can induce gallbladder contraction, while somatostatin (SS) and vasoactive intestinal peptide (11) can inhibit gallbladder contraction. These hormones interact with each other and eventually lead to CL development.

### Changes in the composition of bile

The development of CL after gastric cancer surgery is closely related to changes in gallbladder physiology and changes in bile stone-causing components (5). Most CL cases after gastric cancer surgery involve bile pigment stones (3) and their formation is mainly related to biliary tract infection and cholestasis. Chijiiwa et al. (22) evaluated the gallbladder bile, bile lipid composition, and bile redness in patients who had previously undergone gastrectomy. The stone-causing difference between calcium and ionized calcium was compared, and it was found that ionized calcium and unconjugated bilirubin were significantly increased. These results showed that gallbladder bile tended to contain pigment stones after gastrectomy were an important factor for CL formation, which may be caused by the elimination of gallbladder contraction by gastrectomies and the inactivation of gallbladder bile as well as the mixture of gallbladder bile and fresh hepatic bile, resulting in hypersaturation of the mucosal surface and increasing the tendency toward salt precipitation and gallstone formation.

### Oddi sphincter dysfunction

The dysfunction of the Oddi sphincter may be related to the hepatobiliary branch of the vagus nerve injury, and also related to the release change of gastrointestinal hormones. Nabae et al. (13) recorded Oddi sphincter motility after vagus nerve transection and found that the base pressure of the Oddi sphincter is significantly reduced, but the amplitude is increased. But after meal ingestion, the contraction of the Oddi sphincter increases, and the frequency slows down. This partly explains the cause of CL after gastric cancer surgery.

**Figure 1:**
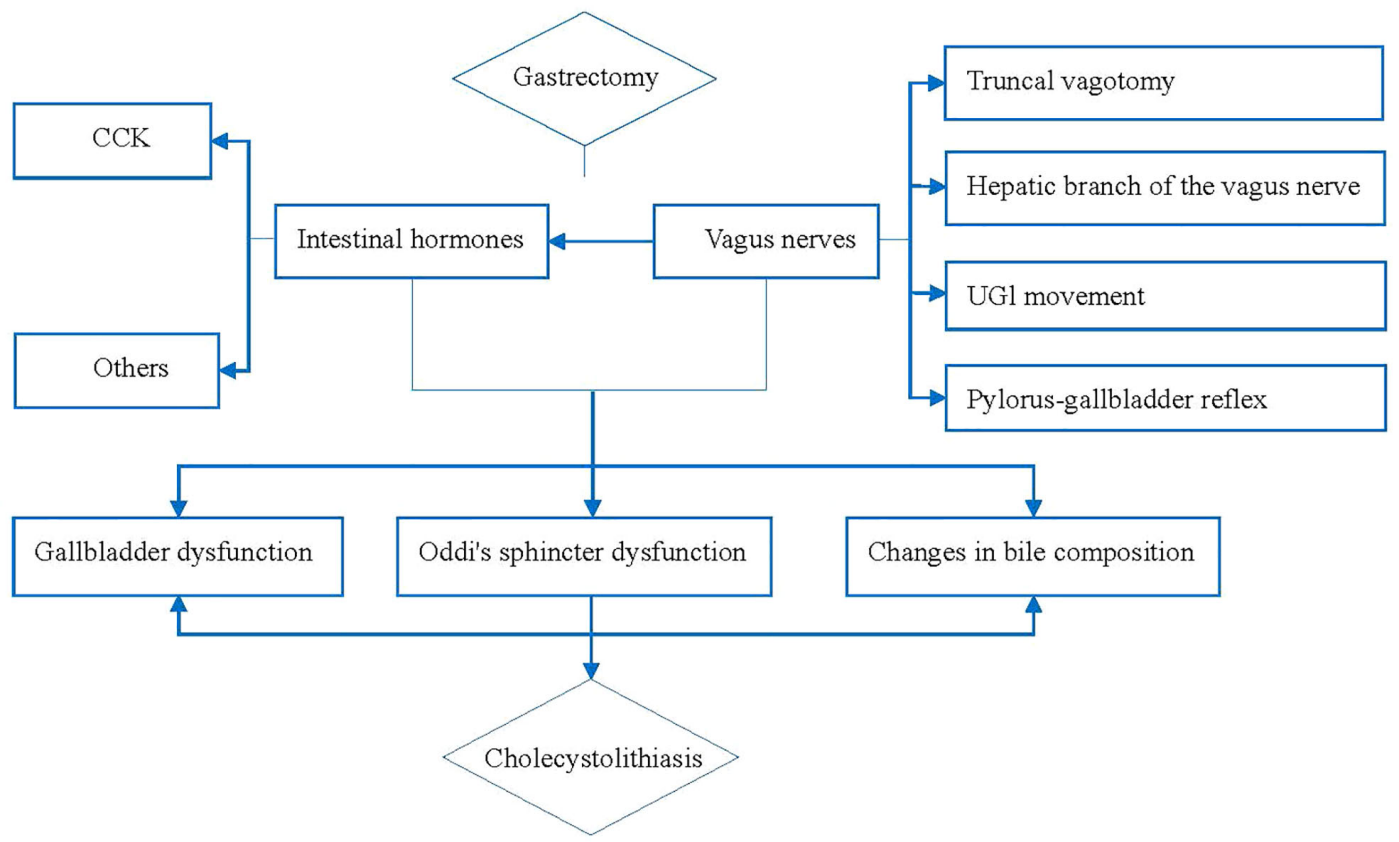
Pathophysiological mechanism of gallstone development after gastric cancer surgery.

In conclusion, vagal nerve injuries, include the vagus trunk, hepatobiliary branch of the vagus nerve, upper digestive tract movement and pyloric-bile duct reflex, changes in the secretion of intestinal hormone and the composition of bile, and Oddi sphincter dysfunction are the main causes and closely related to CL development after gastric cancer surgery. Based on the above evidence, we summarized the mechanism as shown in **Figure 1**. We believe that injury or resection of the vagus nerve or changes in intestinal hormone secretion can lead to physiological dysfunction of the gallbladder (23) and Oddi sphincter, and the lithogenic components in the bile are also changed, ultimately leading to CL (12, 14, 16, 18, 20, 24).

## Risk factors for CL development after gastric cancer surgery

CL development is related to many factors, and we summarized these by consulting multiple studies (see **Table 1**).

**Table 1:** The risk factors for cholecystolithiasis development after gastric cancer surgery.

Preoperative	Intraoperative	Postoperative
Sex	Type of gastrectomy	Total parenteral nutrition
Age	Digestive tract reconstruction	Weight loss
BMI	Extent of lymph node dissection	Immobilization
Percentage decrease in BMI	Jejunum climbing length	Pathological type
Tumor location	Blood supply	Postoperative complications
Clinical stage	Perioperative blood transfusion	Infectious febrile dehydration
Diabetes	Combined evisceration	Immunity
Triglycerides	Laparotomy	Chemotherapy and radiotherapy
Cirrhosis	Inflammation and Machinery	Analgesic
Hepatitis		Gallbladder volume
Coagulation function		Inflammation and machinery
Liver function		Bacterial translocation
Cardiovascular diseases		
Chemotherapy and radiotherapy		

### The type of gastrectomy

Studies have shown that the type of gastrectomy is an independent risk factor for CL (3), especially total gastrectomy (5, 25, 26) and distal gastrectomy (13). The incidence of CL after total gastrectomy is significantly higher than that after other surgical methods, ranging from 6% to 71%, and the onset time is early (4, 5, 9, 26). Jun et al. (4) retrospectively studied 2480 patients undergoing gastrectomy, and the results showed that the incidence of CL after total gastrectomy (10%) was significantly higher than that after subtotal gastrectomy (3%) (P<0.001). A national retrospective cohort study in Korea found that CL was most common after total gastrectomy (6.6%), followed by proximal gastrectomy (5.4%), distal gastrectomy (4.8%), and pylorus-sparing distal gastrectomy (4.0%) (P < 0.001). Multivariate analysis showed that total gastrectomy is an independent risk factor for CL development after gastric cancer (26).

However, Park et al. (8) retrospectively reported that 110 (11.2%) of 979 patients who underwent distal gastrectomy for cancer developed CL after surgery. CL occurred in 32 (13.9%) of 230 patients who underwent total gastrectomy and in 6 (9.7%) of 62 patients who underwent proximal gastrectomy after gastric surgery. CL occurred in 2 (15.4%) of the 13 patients who received PPG, but the difference was not statistically significant (P=0.634), indicating that the type of gastric cancer surgery was not an influencing factor for the development of postoperative CL.

Although, there are conflicting evidences on the relationship between the type of gastrectomy and CL development after gastric cancer. We analyzed that the most likely reason is that the interference of other risk factors is not completely eliminated in the different studies. We still believe that the type of gastrectomy is one of the important risk factors for CL.

### Reconstruction of the digestive tract

The reconstruction of the digestive tract during gastric cancer surgery is closely related to the formation of gallstones. Jun et al. (4) studied 2480 patients undergoing gastric cancer surgery; 128 patients (5.2%) developed CL after surgery, of which Roux-en-Y reconstruction accounted for 60 cases (46.9%), which had a higher risk than Billroth I and Billroth II (P < 0.001). Paik et al. (5) confirmed that the incidence of CL after B2 is higher than that after B1, possibly because the change in the CCK secretion pattern leads to weakened gallbladder systolic function and a high incidence of CL. Studies have shown that nonphysiological reconstruction is an important risk factor for the development of postoperative CL (5, 8) and that this incidence of CL is significantly higher than that after physiologic reconstruction (4), especially with Roux-en-Y (4, 8) and Billroth II (5). The high incidence of CL in the Roux-en-Y procedure can be explained by the extent of gastrectomy and duodenal dissection. Therefore, from above evidences, we believe that Roux-en-Y reconstruction was an independent risk factor for CL development after gastric cancer surgery.

### Degree of lymph node dissection

Thorough lymph node dissection (D2 lymph nodes and enlarged lymph nodes dissection), especially the dissection of hepatoduodenal ligament lymph nodes(No.12), is closely related to the occurrence of postoperative CL. Akatsu et al. (27) studied 805 patients with gastric cancer who underwent D1 (n=490) and D2 (n=315) lymph node dissection. During the follow-up, 102 (12.7%) patients developed CL. D2 lymph node dissection was higher than D1 lymph node dissection (17.8% vs. 9.4%, P = 0.001). Fukagawa et al. (2) found that 173 (25.7%) of 672 patients with gastric cancer who underwent lymph node dissection developed CL after surgery and that enlarged lymph node dissection was a significant risk factor for CL development after surgery (P < 0.001: D1+α vs. D2+α; P < 0.01: D2 vs. D2+α). This may be No.12 lymph nodes are adjacent to the gallbladder and pyloric branches of the vagus nerve that innervate the gallbladder and the common bile duct. During the dissection of No. 12 lymph nodes, the hepatoduodenal ligament is completely “skeletal” (4), and lead to the injury of hepatobiliary branch of the vagus nerve (28). The above studies confirmed that hepatoduodenal ligament lymph node dissection is the most important independent risk factor for postoperative CL development.

### Weight

Paik et al. (5) reported that BMI decline ≥4% (kg/m^2^) was an independent risk factor for CL development after gastric cancer surgery. Park et al. (8) reported that weight cycling (weight loss and weight gain) and large weight fluctuations are risk factors for the development of CL, and concluded from multivariate analysis that obesity is an independent risk factor for the development of CL after gastric cancer surgery. In general, all the proof suggesting a link between a high BMI value and a higher incidence of CL after gastric cancer surgery. Weight loss after gastric cancer surgery leads to the mobilization of cholesterol reserves. Without the activation of gallbladder bile, cholesterol and bile are supersaturated, thus leading to CL. In addition, excessive obesity may increase cholesterol secretion by the liver, which is the key reason for the formation of cholesterol stones.

### Liver function

Nakamura et al. (29) analyzed 698 patients with gastric cancer who underwent surgical treatment and found that abnormal liver function after surgery is related to the development of CL. Lee et al. (30) reported that a high preoperative serum total bilirubin value is an important independent risk factor for the development of CL after gastric cancer surgery. The reason may be that a high level of bilirubin easily leads to gallbladder crystallization, especially the formation of brown or melanin stones. Studies have reported a positive correlation between high triglycerides and gallstone disease. Triglyceride synthesis is stimulated by insulin; So it is concluded that the etiology of CL after gastric cancer surgery may be related to two variables, namely, triglycerides and insulin (31).

### Sex

Most studies have reported that male sex is a risk factor for the development of CL after gastric cancer surgery (5, 8, 26). In a nationwide study conducted in South Korea, Seo et al. (26) reported that men had a higher incidence of CL after surgery than women (5.8% vs. 4.1%, P <0.001) and revolved that male sex was an independent risk factor for CL after gastric cancer.

### Age

Lai et al. (32) reported that elderly patients (aged over 60) are at high risk for the formation of gallbladder stones after gastric cancer surgery. Similarly, Seo et al. (33) reported that older patients (60~89 years old) have a higher incidence of CL (6.1% vs. 4.3%, P < 0.001) than younger patients (30~59 years old) and that advanced age (60~89 years). This may be due to increased bile secretion and intestinal absorption of cholesterol with age, reduced liver synthesis and secretion of bile salts, and reduced gallbladder contractility, all of which increase the susceptibility to cholesterol stones. In terms of genetics, elderly patients may exhibit an enhanced prevalence of the Lith (gallstone) gene, which would promote the formation of gallstones after gastric cancer (34). It was revolved that age is an independent risk factor for CL development after gastric surgery.

### Diabetes

Paik et al. (5) studied 1480 patients who underwent gastrectomy without CL before surgery and concluded that diabetes is an independent risk factor for CL development after gastric cancer surgery. The effect of diabetes on CL is multifactorial, and the mechanism may be that diabetes leads to neuritis and neuromyopathy. In diabetic patients, oxidative stress from low heme oxygenase-1 levels increases, and insulin levels decrease. Insulin-like growth factor-1 signal transduction leads to loss of Cajal interstitial cells, leading to abnormal gallbladder emptying and promoting CL development (35).

### Gallbladder volume

Rieu et al. (23) conducted a prospective study to determine the effects of partial gastrectomy without vagus nerve transection on postprandial gallbladder contraction, CCK secretion, and pancreatic polypeptide PP and found that the basal gallbladder volume was larger after surgery than before (P < 0.02). Portincasa et al. (36) divided the study subjects into a gastrectomy group and a control group and concluded that the gastrectomy group had a larger gallbladder volume during fasting than the control group and that the gastrectomy patients’ gallbladders emptied faster after a meal. In these patients, the postoperative fasting gallbladder volume increased over time. After eating the experimental meal, the patients’ feelings of satiety, abdominal distension and epigastric pain were significantly higher than those in the control group. Therefore, it is believed that the increased gallbladder volume during fasting may lead to cholestasis, which may play an important role in the pathogenesis of CL development after gastrectomy.

### Others

Many other factors, such as bacterial translocation (BT), surgical methods(laparoscopic vs open) (8), parenteral nutrition, dietary lifestyle changes, length of the jejunal loop, postoperative complications (29), combined organ resection, blood supply, perioperative drugs, are also thought to be involved in gallstone formation after gastric cancer surgery.

From the above evidences, we found that the formation of gallstone after gastric cancer surgery is the result of the joint action of multiple factors and mechanisms. Single factor can hardly explain the cause and mechanism of CL development. Previous studies have identified many independent risk factors for postoperative CL, but have not elucidated their intrinsic association of them. Mechanistic research is complex and lengthy and do not provide useful therapeutic value. Instead of wasting time and resources on etiology and mechanism research, it is better to work directly on treatments.

## Treatment of CL after gastric cancer surgery

Studies have reported that preserving the vagus nerve that innervates the gallbladder during gastric cancer surgery (37), and pyloric-preserving gastrectomy (PPG) (38), and prophylactic use of drugs such as erythromycin (39) and ursodeoxycholic acid (39, 40) can reduce the development of cholecystolithiasis, but the clinical effect is controversial. Which strategies are the most appropriate treatments for CL after gastric cancer surgery?

### Watchful waiting

Studies have reported that most postoperative CL cases associated with gastric cancer are asymptomatic (3, 9), so most scholars support watchful waiting as the most reasonable treatment method (41), but there are potentially fatal hazards, such as inducing acute pancreatitis, acute cholangitis and gallbladder cancer, with high mortality.

### Vagal-nerve-sparing gastrectomy

Studies have reported that retaining the vagus nerve that innervates the gallbladder during gastric cancer surgery can ensure the normal function of the gallbladder and significantly reduce the incidence of gallbladder stones (13, 19); however, there are also studies showing that some patients (10.1%) still relapse after surgery (19). A prospective case–control study by Wang et al. (42) compared the vagus nerve-sparing group (h-DG, n=85) and the dissected nerve group (s-DG, n=238) under laparoscopic distal gastrectomy and the vagus nerve preserving group (h-PPG, n=123) and dissected nerve group (s-PPG, n=21) under laparoscopic pylori-preserving gastrectomy. The results showed that the 3-year cumulative incidence of CL in the h-DG group was significantly lower than that in the s-DG group (2.7% vs. 14.6%, P=0.017). Similarly, this incidence in the h-PPG group was significantly lower than that in the s-PPG group (1.6%). vs. 12.9%, P=0.004). It suggested that both vagal-nerve-sparing gastrectomy and PPG can significantly reduce the incidence of CL development after gastric cancer surgery (38, 42). However, recent studies have suggested that PPG has no effect on the development of CL (10, 13, 26). In our view, vagal-nerve-sparing gastrectomy is complicated, which is not conducive to promotion and implementation. In addition, the formation of CL after gastric cancer surgery is multi-factor, and the effect of prevention from a certain factor is not certain. So, this approach is not valuable to be recommended.

### Medical prevention

Some studies have reported that prophylactic use of drugs such as erythromycin (15), ursodeoxycholic acid (39, 40), exogenous CCK-8, cisapride, motilin, domperidone, cimetidine, opioid antagonists, naloxone, and aspirin can reduce CL development after gastric cancer surgery. A prospective multicenter randomized controlled clinical trial conducted by Lee et al. (40) randomly divided 465 patients who underwent gastric cancer surgery into 151 patients who received 300 mg ursodeoxycholic acid (UDCA), 164 patients who received 600 mg UDCA, and 150 patients who received placebo. The results showed that after 12 months, the incidence of CL was 5.3% (8/151) in the 300 mg group, 4.3% (7/164) in the 600 mg group, and 16.7% (25/150) in the placebo group. Compared with the placebo group, the results indicating that 12 months of UDCA after gastric cancer surgery can significantly reduce the incidence of CL. However, studies have shown that gallstones after gastric cancer are dominated by bile pigment stones (3). Oral stone-dissolving drugs are less effective and require a long treatment period. So, the clinical effect of medical is not satisfactory. Long-term medication will increase the physical, psychological and economic burdens on patients.

### Secondary surgery

Cholecystectomy is the “gold standard” for the treatment of CL. With the improvement of minimally invasive surgery concepts and techniques, laparoscopic cholecystectomy (LC) and endoscopic retrograde cholangiopancreatography (ERCP), endoscopic sphincterotomy (EST), endoscopic papillosphincter balloon dilatation (EPBD), percutaneous hepatobiliary drainage, and percutaneous puncture drainage have gradually been applied to patients with a history of gastric cancer surgery. Minimally invasive surgery (represented by LC and ERCP+EST) can significantly shorten the disease course and relieve patients’ pain and is considered to be safe and effective (33). However, some studies have shown that cholecystectomy after gastric cancer surgery increases the difficulty of surgery, mortality, and the cost of surgery (9, 16, 43, 44). Application of ERCP is technically difficult due to anatomical changes, such as after physiologic reconstruction (4), especially with Roux-en-Y (4, 8) and Billroth II (5).

At present, there is no unified guideline for the selection of CL treatment strategy after gastric cancer surgery. Due to the shortcomings and deficiencies of various treatment methods, the treatment effect is not satisfactory. As a new treatment strategy, PC is expected to become the most valuable treatment for CL after gastric cancer in the future. Surgeons have been devoted to the study of PC during gastric cancer surgery.

## Proposed background of the PC concept

### Incidence of CL after gastric cancer surgery

Most studies have reported that the incidence of CL within 5 years after gastric cancer surgery is as high as 15%-25%, with an average of 17%. In previous studies, the rate was as high as 47%-60% (33). Liang et al. (3) and Bernini et al. (45) reported that the incidence of CL after gastric surgery showed an increasing trend over time and had not yet reached an equilibrium level. Bencini et al. (3) reported that for patients with gastric cancer who survived 60 months after surgery, the incidence of postoperative CL increased by 18.5%. However, some studies reported that the incidence of CL after gastric surgery was less than 10% (4, 5, 9, 46, 47), and the incidence of cholangitis or cholecystitis in patients with CL after gastric cancer surgery was 20.3%, which was almost the same as that in the general population (9).

### Interval time for CL development after gastric cancer surgery

Liang et al. (9) and Fukagawa et al. (2) both reported that 64.7% of CL cases developed within 1 year after gastric surgery, and Murata A et al. (48) confirmed that the earlier the development of CL after gastric cancer surgery, the greater the threat to the prognosis and quality of life of patients.

### Complications related to CL after gastric cancer surgery

Acute postoperative cholecystitis, defined as occurring during the same hospital stay or within 30 days after surgery (49), is a serious complication, especially for patients who have recently undergone gastrectomy (50–52); it is difficult to diagnose and has mortality rates as high as 10-50% (52). **Table 2** show all the studies related to the acute postoperative cholecystitis and revolved that PC can minimize the risk of acute postoperative cholecystitis (53) and avoid the formation of gallstones (2, 3, 11).

**Table 2:** Acute cholecystitis after gastrectomy for cancer.

Author	Patients(n)	Surgery	AC(n)	Character	FT (day)	Surgical (n)	Mortality
Takahashi (44)	1096	gastrectomy	7(0.6%)	NC	20 (5-70)	7 (0.6%)	4 (0.4%)
Oh (52)	8033	gastrectomy	5 (0.06%)	NC	14 (2-31)	5 (0.06%)	0%
Ito (51)	190	gastrectomy	24(12.6%)	NC	ns	6 (3.2%)	0%
Wu (50)	288	gastrectomy	9 (3.1%)	NC	ns	7 (2.4%)	2 (0.6%)

AC, acute cholecystitis; NC, noncalculous cholecystitis; FT, formation time; ns, not stated.

Stones is also a common complication related to CL. Studies have shown that most CL cases occurring after gastric surgery involve multiple (72.5%) stones that are less than 10 mm in diameter (79.4%) (27); small stones are an independent risk factor for pancreatitis (54), which will increase the incidence of biliary complications. Bencini et al. (3) reported that 12.3% of patients ain the standard gastric surgery (SS) group had abnormal biliary tracts after surgery, while only 1.5% of patients in the PC group had common bile duct dilation (CBD) after surgery, and the difference was statistically significant. (8/65 vs. 1/65, P= 0.033). This suggests that PC can reduce the biliary tract related complications.

### Secondary operation related to CL after gastric cancer surgery

Studies have reported a high proportion of secondary surgical intervention due to biliary tract diseases after gastrectomy, and frequent biliary colic and cholecystitis are the main causes of subsequent cholecystectomy (32). Moreover, the upper abdominal inflammatory adhesions caused by previous gastrectomy make the subsequent operation difficult (9, 16, 33), and the incidence of surgical complications, common bile duct injury, mortality(20.0-57.0%) (51), medical expenses, average hospital stay, medical resources, conversion rate to laparotomy(10-50%) (54), and operation time of subsequent cholecystectomy after gastric cancer surgery increases, especially in emergency operations (9, 11, 16, 44). Gillen et al. (33) reported that the surgical risk of subsequent cholecystectomy after gastric cancer surgery is 15 times higher than that of PC during gastrectomy for cancer and the incidence of surgical complications, common bile duct injury, mortality, medical expenses, average hospital stay, medical resources, conversion rate to laparotomy, and operation time of subsequent cholecystectomy after gastric cancer surgery increases significantly, especially in emergency operations (9, 11, 16). Studies have reported mortality rates of subsequent cholecystectomy as high as 20.0-57.0% (51), and the laparoscopic conversion rate is 10-50% (54). The reasons may be related to a patient’s advanced age, poor physical condition (44) and difficulty in managing the inflammatory gallbladder in the surgical field due to prior gastrectomy and lymph node dissection (9).

Gastrectomy will result in secondary bile duct stones, and the change in anatomical structure after gastrectomy will increase the difficulty of reaching the duodenal papilla during elective surgery (5), especially when duodenal reconstruction (Billroth II or Roux-en-Y (45)) is applied. Paik et al. (5) reported that only 6 of the 20 patients with choledocholithiasis after gastrectomy successfully underwent ERCP. For patients with a history of gastrectomy, subsequent cholecystectomy is often accompanied by a risk of damage to the bile duct and surrounding tissues (55), among which bile duct injury is the most serious complication and has a high mortality rate.

Gastrectomy combined with cholecystectomy is a mostly laparoscopic surgery, as surgeons have gained experience in laparoscopic surgery and exceeded the “learning curve” (56). The safety and feasibility of surgery have been greatly improved (57). Combined gastric cancer surgery has been greatly improved. Therefore, some scholars believe that if PC does not increase serious surgical complications, it would be better than subsequent cholecystectomy after gastric cancer surgery (43).

### Quality of life and laparoscopic techniques

In the surgeon’s clinical work, an effective assessment of a patient’s quality of life can affect the decision of whether to operate. Fukagawa et al. (2), Bernini et al. (45) and Wu et al. (50) all reported that PC could improve the quality of life of patients who survived. Mentes et al. (58) used the gastrointestinal quality of life score to evaluate the effect after cholecystectomy and concluded that PC during gastric cancer surgery at least does not reduce postoperative quality of life.

Due to the high incidence and short interval time for CL development after gastric cancer surgery, additional, PC can minimize the risk of acute postoperative cholecystitis, it also can avoid the difficulty of secondary surgical intervention after gastrectomy and surgical risk and the high incidence of surgical complications after the gastrectomy, these advantages provide the best opportunity for using PC.

However, it has been reported that almost all biliary abnormalities after gastrectomy were detected by ultrasound 4.5 years later (2, 3, 11). The 3-year mortality rate after gastrectomy was the highest, which would suggest that PC is not necessary for most people, since for some patients who undergo surgery for gastric cancer, gallstones rarely form before the end of their lives. Studies also have reported that almost 90% of CL cases developing after gastrectomy are asymptomatic (2, 5, 10), and the longer the time from surgery, the less likely patients are to develop symptoms (11). Hence, few patients have symptoms after surgery (0.6% to 4.6%) or require additional treatment (0.4% to 4.6%) (2, 3, 5, 9, 10, 32, 54) (**Table 3**). Fukagawa et al. (2) studied 173 patients who developed CL after gastric cancer surgery. Among the asymptomatic group (161 cases), 77.0% (124 cases) had no change in the size or number of gallstones. Paik et al. (5) studied 1480 patients who underwent gastric cancer surgery, and the results showed that the incidence of postoperative choledocholithiasis was only 1.4% (20 patients), and its development was only related to CL, suggesting that the effect of secondary choledocholithiasis after gastric cancer surgery was negligible. Additional, with improvements in surgical techniques and medical equipment, some studies have reported that subsequent laparoscopic cholecystectomy is safe and reasonable for patients with a previous gastrectomy history (4, 31, 42, 44, 49, 59–62) (**Table 4**).

**Table 3:** CL development after gastric cancer surgery is rarely symptomatic and requires surgical intervention.

Author	Gastrectomy(n)	CL	Symptomatic CL	Surgical
Liang (9)	17,325	1280 (7.4%)	ns	560 (3.2%)
Bencini (3)	65	8 (12.3%)	3 (4.6%)	3 (4.6%)
Paik (5)	1480	106 (7.2%)	9 (0.6%)	9 (0.6%)
Fukagawa (2)	672	173 (25.7%)	12 (1.8%)	12 (1.8%)
Lai (32)	197	30 (15.2%)	9 (4.6%)	9 (4.6%)
Kobayashi (10)	749	86 (11.4%)	6 (0.8%)	3 (0.4%)
Akatsu (27)	805	102 (12.7%)	15 (1.9%)	13 (1.6%)

CL, cholecystolithiasis; ns, not stated.

**Table 4:** Subsequent cholecystectomy after gastrectomy is safe and feasible.

Author	Groups	Conclusion
Gillen (33)	PC vs. SC	The complication rate and mortality in the SC group were lower than those in the PC group.
Kimura (47)	Cholecystitis and/or cholangitis	Treatment of postoperative cholecystitis and/or cholangitis is effective and does not increase complications or length of hospital stay.
Kim (63)	OCL vs. LCL	Compared with OC, LC for gallstones after gastric cancer surgery results in earlier recovery of diet, shorter hospitalization times and less incidence of complications.
Kwon (64)	CLPG	LC after gastric cancer surgery does not increase the operative time, length of hospital stay, postoperative complications and time to complete normal activities.
Zhang (65)	CLPG vs. CLNPG	Compared with CLNPG, CLPG does not increase the blood loss, conversion rate, intraoperative bile duct injury rate, diet recovery time, and postoperative hospitalization time.
Lai (32)	SC	The incidence of complications and mortality of SC are zero.
Inoue (49)	SC	Cholecystectomy has the lowest mortality and is the optimal treatment for acute cholecystitis after gastric cancer surgery.
Jun (4)	OCL vs. LCL	The operation time and hospitalization time of LCL are shorter than those of OCL.
Sasaki (66)	CLPG vs. CLNPG	CLPG increases the incidence of choledocholithiasis and operative time but does not increase the blood loss, conversion rate, complication rate, recovery time to diet, or postoperative hospital stay

PC, Prophylactic cholecystectomy; CL, Cholecystolithiasis; OC, Open cholecystectomy; LC, Laparoscopic cholecystectomy; SC, Subsequent cholecystectomy; OCL, Open cholecystectomy after gastric cancer surgery; LCL, Laparoscopic cholecystectomy after gastric cancer surgery; CLPG, Cholecystectomy in patients with a prior history of gastrectomy; CLNPG, Cholecystectomy in patients without a prior history of gastrectomy.

From the above evidences, we can found that most of the evidences support the implementation of PC, although there were some arguments to the contrary. Some scholars believe PC is safe and effective (**Table 5**) (15, 20, 21, 27, 54), while others disagree (**Table 6**) (3, 5, 10, 29, 30). Moreover, certain scholars believe that the feasibility of PC should be individualized (9). Should PC be used as a routine treatment? We reviewed lots of studies supporting or against PC.

**Table 5:** Studies supporting PC during gastric cancer surgery.

Author	Patients(n)	Gallbladder	Conclusion
Thompson	56	Any gallbladder	(1) PC adds minimal morbidity. (2) The majority of patients with CL after gastrectomy become symptomatic and require secondary surgery, and secondary surgery increases the morbidity.
Saade	109	Asymptomatic CL	(1) PC adds minimal morbidity. (2) The number of secondary cholecystectomies after gastrectomy is large.
Watemberg (67)	4072	Any gallbladder	Complication and mortality rates increase significantly and dreadfully when the gallbladder is left *in situ* after gastrectomy.
Jeong (43)	400	Any gallbladder	PC is safe and feasible in patients with both early gastric cancer and gallbladder disease.
Bernini (68)	130	Any gallbladder	PC added no extra perioperative morbidity, mortality or costs to the sample included in the study.
Lai (32)	445	Asymptomatic CL	PC is not associated with increased surgical morbidity or mortality, and has no significant effect on overall survival.
Miftode (16)	206	Any gallbladder	PC can be safely performed during gastrectomy and thus prevents complications at a later stage.
Murata (69)	14,006	Any gallbladder	PC does not affect the prognosis of patients undergoing gastric cancer surgery.

PC, Prophylactic cholecystectomy; CL, Cholecystolithiasis.

**Table 6:** Research against PC during gastric cancer surgery.

Author	Patients(n)	Conclusion
Kobayashi (10)	749	(1) Hepatoduodenal ligament lymph node dissection, total gastrectomy and duodenum exclusion are risk factors for the development of CL after gastric cancer surgery. (2) The majority of CL cases are asymptomatic (93%), and less than 0.5% of patients need cholecystectomy.
Gillen (33)	3735	The incidence of CL after gastrectomy is low (6%), and selective cholecystectomy is safe.
Shim (53)	–	(1) PC is not ethical. (2) CL after gastric cancer surgery rarely requires surgery. (3) Minimally invasive surgery can effectively treat CL after gastric cancer surgery. (4) Digestive problems occur after PC.
Paik (5)	1480	(1) Advanced age, diabetes, surgical methods, male sex and decreased body mass index are high risk factors for the development of CL after gastric cancer surgery. (2) PC should not be routinely recommended for use during gastric cancer surgery.
Bencini (3)	130	PC has no significant effect on the natural course of gastric cancer patients.

PC, Prophylactic cholecystectomy; CL, Cholecystolithiasis.

## Studies supporting PC

Some studies have reported that PC during gastric cancer surgery is safe, effective and reasonable when compared with SS or subsequent cholecystectomy after gastrectomy because PC during gastric cancer surgery does not increase postoperative complications, gallbladder-related complications, postoperative pulmonary complications (POPCs), biliary complications, surgical complications, nonsurgical complications, complications related to laparoscopy, overall mortality, mortality within 30 days after surgery, in-hospital mortality, average operation time, postoperative hospital stay, postoperative gastrointestinal function recovery time, parenteral nutrition time, enteral nutrition time, overall morbidity, intraoperative blood loss, risk of anesthesia or hospital costs (16, 20, 21, 25, 26, 43).

Jeong et al. (43) retrospectively studied 400 patients who underwent laparoscopic-assisted gastrectomy for early gastric cancer, and found that PC may extend the average operation time by approximately 15 minutes, but it has no effect on the effect of surgery. In all patients with early gastric cancer and gallbladder disease, combined intraoperative cholecystectomy for gastric cancer seems to be safe and feasible.

Bernini et al. (45) analyzed a multicenter randomized controlled clinical trial of 130 patients with gastric cancer, and found that PC during gastric cancer surgery will not increase morbidity, mortality, or hospitalization costs.

Lai et al. (32) retrospectively analyzed 445 patients who received gastrectomy. Among them, the combined cholecystectomy group (n=58) and the simple gastrectomy group (n=387) were analyzed. There were no significant differences in the mortality rate (3.4% vs. 3.1%), complication rate (24.2% vs. 22%) or 5-year survival rate (61% vs. 63%) (P > 0.05). At the same time, there were no significant differences in hospital stay, conversion rate or operation time. Therefore, the authors believe that PC can be considered for patients with asymptomatic CL before surgery. Miftode et al. (16) reported that the mortality rate of patients undergoing cholecystectomy after gastrectomy was 63.63% (7/11) and that the mortality rate of patients undergoing PC was 4.92% (3/61); moreover, patients undergoing PC had fewer postoperative complications. Therefore, the authors believe that PC is safe and feasible for reducing postoperative complications and secondary surgery.

A Japanese study based on the National Administrative Database (70) explored the prognosis of laparoscopic gastrectomy combined with cholecystectomy, including 14,006 patients with gastric cancer from 744 hospitals. The subjects were divided into a combined cholecystectomy group (n=1484) and a simple gastrectomy group (n=12,522). The results showed that PC did not increase laparoscopic-related complications (OR, 1.02; 95% confidence interval [CI], 0.84-1.24; P=0.788), mortality during hospitalization (OR, 1.16; 95% CI, 0.49-2.76; P=0.727) or hospital stay (unstandardized coefficient, 0.37 days; 95% CI, -0.47 to 1.22 days; P = 0.389). However, PC significantly increased the cost of hospitalization (unstandardized coefficient, $1256.0 (95% CI, $806.2-$1705.9; P <0.001). The author believes that although the medical expenses during hospitalization have greatly increased, laparoscopic gastrectomy combined with cholecystectomy will not affect the patient’s prognosis.

Tan et al. (46) retrospectively analyzed 1,753 patients with gastric cancer who received subtotal gastrectomy or total gastrectomy and divided them into the combined cholecystectomy group (n=62) and the simple gastrectomy group (n=1,691). The results showed that there was no statistically significant difference in mortality and complication rates between the two groups (8.1% vs. 8.9%). Thus, the authors believe that PC will not increase postoperative mortality and morbidity.

All these studies have proved that PC during gastric cancer surgery is safe, effective and reasonable when compared with SS or subsequent cholecystectomy after gastrectomy because PC during gastric cancer surgery does not increase postoperative complications, gallbladder-related complications, postoperative pulmonary complications (POPCs), biliary complications, surgical complications, nonsurgical complications, complications related to laparoscopy, overall mortality, mortality within 30 days after surgery, in-hospital mortality, average operation time, postoperative hospital stay, postoperative gastrointestinal function recovery time, parenteral nutrition time, enteral nutrition time, overall morbidity, intraoperative blood loss, risk of anesthesia or hospital costs (16, 20, 21, 25,7,^27^, ^46^).

## Research against PC

Gillen et al. (33) studied 3,735 patients who underwent upper gastrointestinal surgery and showed that the mortality rate in the PC group was 0.95%, compared with 0.45% in the preoperative or postoperative cholecystectomy group, with a significant difference (95% CI, 0.54–1.49%; I^2^ = 28%). Moreover, the incidence of complications in the PC group was higher than that following subsequent cholecystectomy. Therefore, it has been proposed that removal of the normal gallbladder during gastric cancer surgery is not recommended.

Bencini et al. (3) evaluated the need for PC during gastrectomy in a multicenter randomized controlled trial. A total of 130 patients with gastric cancer were enrolled and divided into a PC group (n=65) and a simple gastrectomy group (n=65). Eight patients (12.3%) in the control group had biliary tract abnormalities (4 cases gallstones and 4 cases cholestasis), and only three (4.6%) were clinically relevant (2 underwent cholecystectomy and 1 case acute pancreatitis). One patient in the PC group had asymptomatic biliary dilatation. The 5-year survival rate in the control group was 60% (95% CI: 47-71%), while that in the PC group was 59% (95% CI: 44-71%). The difference was not statistically significant (log-rank test: P=0.697). Similarly, for early gastric cancer (AJCC stage I or II), there was no statistically significant difference in 5-year survival between the PC group (76% (95% CI: 57-87%) and the control group (77% (59-88%)). There was no statistically significant difference in CL survival between the two groups (P = 0.267). To prevent secondary CL, 1 in 32.5 patients needed to be treated with PC. The authors believe that although PC is safe and can effectively prevent the occurrence of CL in the long term, PC cannot effectively improve the patients’ natural course of disease. Most CLs formed after gastric cancer surgery are asymptomatic and delayed, and the actual proportion of surgical intervention due to symptoms is 1:32.5. Such a low percentage does not allow PC to become a routine procedure.

Paik et al. (5) analyzed 1,480 patients who underwent gastrectomy; the results showed that the incidence of CL was low (7.2%, 106/1480), cholecystitis was only found in 9 patients (0.6%), and the incidence of choledocholithiasis was 1.4% (20/1480) Therefore, the authors did not recommend PC as a routine operation. Kobayashi et al. (10) retrospectively analyzed 749 patients who underwent gastric cancer resection. The results showed that the cumulative incidence of CL at 5 and 10 years after surgery was 13.6% and 22.1%, respectively, and 93% (80/86) of the patients were asymptomatic; only 0.4% of patients underwent cholecystectomy, so the authors believed that PC was unnecessary. Shim et al. (53) believed that CL development after gastric cancer surgery rarely requires surgery, and that minimally invasive surgery can effectively treat postoperative CL. In addition, PC is unethical, and a series of digestive problems can occur after cholecystectomy.

Digestive problems after PC during gastric cancer surgery are worth discussing (53). These include chronic diarrhea syndrome (71), diarrhea syndrome after laparoscopic cholecystectomy (postlaparoscopic cholecystectomy diarrhea, PLCD) (71), bile reflux gastritis and esophagitis (59, 72), Mirizzi syndrome (60), chronic pain (61) and secondary common bile duct cholelithiasis (62). Yueh et al. (71) reported that diarrhea occurred in 25.2% of patients 1 week after laparoscopic cholecystectomy and that 5.7% of patients developed diarrhea after 3 months. The incidence of postcholecystectomy syndrome (PCS) after cholecystectomy is 10 to 40% (3, 60), and approximately 5% of patients have chronic pain without an obvious cause (61). Patients receiving cholecystectomy are at risk for chronic postoperative pain, with an incidence ranging from 10 to 40% (62). At present, there are few reports about digestive system diseases after gastric cancer surgery, but theoretically, the abovementioned digestive system diseases will have a great negative impact on the postoperative quality of life of patients, which should be considered.

To date, most studies have discussed the short-term endpoint of the safety of PC during gastric cancer surgery (incidence of complications, duration of surgery, postoperative hospital stay, etc.) (45). However, it was concluded that while PC during gastrectomy for gastric cancer was safe for approximately 5 years of follow-up, the long-term effect was inconclusive (68).

The incidence of CL after gastrectomy was reported to be transient (40), and some cases of CL disappeared during follow-up (73). Inoue et al. (19) and Takahashi et al. (44) both reported that gallbladder contraction recovered to near the preoperative level within 3 months after gastrectomy, and the cholestasis development rate gradually decreased. Inoue et al. (28) reported that in 64.3% of patients, CL formed within 1 year after surgery, and only 19.3% of patients developed CL in the second year after surgery, which may be related to the recovery of gallbladder contraction function 1 year after gastric cancer surgery.

Some scholars believe that PC performed during gastric cancer surgery is inconsistent with the principles of ethics and surgery (53, 68). Both Liang et al. (9) and Cabarrou et al. (6) reported that PC increased medical costs and workload. In recent years, litigation and malpractice claims have raised issues with the removal of normal organs to avoid any benign disease, such as the removal of normal gallbladders (3, 6, 45).

Due to some of the studies reported that the incidence of complications in the PC group was higher than that following subsequent cholecystectomy, the actual postoperative proportion of surgical intervention in a low percentage, ethical issues, and a series of digestive problems can occur after cholecystectomy, all these studies do not recommend PC to become a routine procedure. We summarized the research evidence for and against PC as in **Table 7**. In fact, with the development of surgical techniques, the adverse outcomes caused by PC will become less and less, and most of them can be avoided.

**Table 7:** Research evidence for and against PC.

Reasons supporting PC during gastric cancer surgery
PC is safe and effective.
PC can prevent secondary surgery related to CL.
PC can reduce complications related to CL.
PC can improve postoperative quality of life.
The incidence of CL after gastric cancer surgery is high.
The interval between CL development after gastric cancer surgery is short.
Preserving the gallbladder during gastric cancer surgery delays the diagnosis of gallbladder disease.
The incidence of secondary surgery after gastric cancer surgery is high, the operation is difficult, and the mortality is high.
Cholecystitis, cholangitis, pancreatitis and gallbladder cancer development after gastric cancer surgery.
The mortality rate of acute cholecystitis (within 30 days) after gastric cancer surgery is high.
Conservative treatment of CL after gastric cancer surgery is ineffective.
Reasons not to support PC during gastric cancer surgery
PC increases biliary complications (especially bile duct injury).
PC increases hospitalization costs and length of stay.
PC reduces postoperative quality of life.
PC increases surgical mortality.
PC increases medical litigation and claims.
PC increases the development of secondary choledocholithiasis after surgery.
A variety of digestive system diseases are formed after PC (postcholecystectomy syndrome, PCS)
PC increases the incidence of chronic pain.
The incidence of CL after gastric cancer surgery is low.
Most CL cases developing after gastric cancer surgery are asymptomatic.
CL after gastric cancer surgery rarely requires surgical intervention.
Secondary cholecystectomy after gastric cancer surgery is safe and effective.
Treatment of CL development after gastric cancer surgery does not increase mortality.
The incidence of CL after gastric cancer surgery is low.
Some CL cases disappear naturally after gastric cancer surgery.
The conservative treatment of CL after gastric cancer surgery is effective.
The long-term efficacy of PC is unclear.
PC does not conform to the principles of surgery and ethics.

PC, prophylactic cholecystectomy; CL, Cholecystolithiasis.

## Indications and contraindications for PC

To facilitate the implementation of PC, we reviewed the relevant evidence as follows and summarized its indications and complications as showing in **Table 8**. Through a series of clinical studies, Sasaki et al. (66), Fukagawa et al. (2), Wu et al. (50), and Gillen et al. (33) suggested that PC should be considered for patients who need enlarged lymph node dissection because such dissection can increase the development of postoperative CL. On the one hand, PC does not affect the postoperative recovery of patients with gastric cancer; on the other hand, it can significantly reduce the biliary tract injury caused by cholecystectomy after gastric cancer. Some scholars believe that PC can be considered for patients who cannot carry out duodenal access preservation (74), especially Roux-en-Y (9) and Billroth II (46), because the risk of CL development after Roux-en-Y reconstruction is significantly higher than that after other procedures, and when CL develops after surgery, ERCP is very difficult to perform. Hauters et al. (31) believe that PC can be considered for total gastrectomy because the incidence of CL after total gastrectomy is higher than that after other surgical procedures, and the development of CL occurs early (4, 5, 7, 26). Tan et al. (46) found that Billroth II and diabetes were independent risk factors for postoperative CL development and elective secondary cholecystectomy, so PC was recommended. Bencini et al. (3) and Jeong et al. (22) believe that PC can improve the quality of life of middle-aged and young patients with early gastric cancer because they have a high life expectancy. Lai et al. (32) believe that PC can be considered in male patients and patients older than 60 years. In addition, Liang et al. (9) reported that PC may be beneficial in non-Asian countries with a higher incidence of CL after gastric cancer.

**Table 8:** Conditions under which PC is recommended or not recommended during gastrectomy for cancer.

PC is recommended during gastric cancer surgery:	
Extended lymph node dissection
D2 lymphadenectomy
D3 lymphadenectomy
Hepatoduodenal ligament lymph node dissection
Nonphysiological reconstruction
Roux-en-Y
Billroth II
Total gastrectomy
Liver function impairment
Diabetes
Advanced gastric cancer
Early gastric cancer
Young and middle-aged people with early gastric cancer
High risk (postoperative cholecystolithiasis)
Male patients and patients over 60 years old
PC is not recommended during gastric cancer surgery:
Receive palliative care
Life expectancy is less than six months
Distal gastrectomy with Billroth I reconstruction
D1 lymphadenectomy
Patient retains gallbladder intention

On the other hand, Watemberg et al. (67) believed that for patients who receive palliative care or whose life expectancy is less than 6 months, there is no need for PC during gastric cancer surgery. In addition, Gillen et al. (33) reported that PC was not routinely recommended during intraoperative D1 lymph node dissection for gastric cancer.

In general, the evidence and reasons in favor of PC are significantly more preponderance, and combined with our own work experience, we are more in favor of PC as a standard strategy for the treatment of gallstones after gastric cancer surgery.

## Conclusions

The formation of CL after gastric cancer surgery is the result of multifactorial and mechanisms. Previous studies have identified many independent risk factors for CL after gastric cancer, but have not elucidated the intrinsic association between them. Due to the shortcomings and deficiencies of various treatment methods, the treatment effect is not satisfactory. As a new treatment strategy, PC has undeniable advantages and is expected to become the standard treatment for CL after gastric cancer in the future. The individualized PC strategy for CL after gastric cancer is the main direction of future research.

## Data availability statement

The original contributions presented in the study are included in the article, further inquiries can be directed to the corresponding author.

## Author contributions

HL study design, collation of results, writing original draft, writing-review, and editing. JL, data collection, collation of results, and writing original draft. WX and XC, data collection and collation of results. All authors contributed to the article and approved the submitted version.

## Funding

The research was supported by the Natural Science Foundation of Gansu Province (Project No. 21JR1RA126).

## Conflict of interest

The authors declare that the research was conducted in the absence of any commercial or financial relationships that could be construed as a potential conflict of interest.

## Publisher’s note

All claims expressed in this article are solely those of the authors and do not necessarily represent those of their affiliated organizations, or those of the publisher, the editors and the reviewers. Any product that may be evaluated in this article, or claim that may be made by its manufacturer, is not guaranteed or endorsed by the publisher.
